# Prevalence of negative emotional eating in middle-aged adults: a systematic review and meta-analysis

**DOI:** 10.1186/s40337-025-01476-8

**Published:** 2025-12-05

**Authors:** Katherine Yuk Ping Sze, Elorm Donkor, Zuyao Yang, Jean H. Kim

**Affiliations:** Jockey Club School of Public Health and Primary Care, The Chinese University of Hong Kong, Prince of Wales Hospital, Shatin, New Territories, Hong Kong SAR, China

**Keywords:** Obesity, Middle-aged, Overeating, Emotional eating, Systematic review

## Abstract

**Background:**

Negative emotional eating (EE) is overeating in response to emotions such as stress. Negative EE is a risk factor for obesity, which is, in turn, a risk factor for many non-communicable diseases (NCDs). While previous research has predominantly focused on younger or student populations, the prevalence and determinants of negative EE in middle-aged adults remain underexplored.

**Methods:**

A systematic search was conducted in Medline, Embase, PsycINFO, Web of Science Core Collection, and Google Scholar for English language studies published from 2000 onwards reporting on the prevalence of negative EE in adults aged 35–64 years. Pooled prevalence estimates and corresponding 95% confidence intervals (CIs) were calculated using random-effects models. Heterogeneity was assessed with the I² statistic. Subgroup analyses were performed by age, sex, country income classification, and assessment instrument.

**Results:**

Of 1,390 identified records, 38 studies including 13,662 participants met the inclusion criteria. The pooled prevalence of negative EE among middle-aged adults was 16% (95% CI: 14%-19%). Prevalence was notably higher among younger middle-aged females compared to males and older individuals. Additionally, negative EE was more common in middle-aged adults residing in high-income countries compared to those in middle-income countries.

**Conclusion:**

The global prevalence of negative EE among middle-aged adults highlights the need for targeted health promotion and behavioral interventions in this age group. Early identification and modification of unhealthy eating behaviors could help mitigate the risk of obesity and NCDs, particularly in high-income countries facing a growing burden of obesity-related health issues.

## Abstract (plain language summary)

This study examined the proportion of middle-aged adults who engaged in negative emotional eating, eating more food than usual in response to negative emotions like stress, sadness, or anxiety. By gathering information from 38 studies with over 13,000 people from around the world, this study found that about one in six middle-aged adults struggle with this kind of eating. Negative emotional eating was most common in women, especially those in their late 30s, and among residents of higher income countries. These results demonstrates that eating in response to negative emotions is not limited to young people, but also affects a sizable proportion of adults in midlife. Overeating in response to negative emotions can increase the risk of becoming overweight and thereby developing health problems like diabetes and heart disease. The results suggest that it is important for health services to help middle-aged adults recognize and manage emotional eating. Early support and simple strategies could help people develop healthier ways to cope with difficult feelings, which may improve both their physical and mental health.

## Introduction

 In 2004, the World Health Organization (WHO) adopted a global strategy for diet, physical activity, and health under Sustainable Development Goal 9 (Good Health and Wellbeing) in recognition of the role that eating behaviors play in the development of non-communicable diseases (NCDs) [1]. Obesity now ranks as the fifth leading cause of premature mortality worldwide, accounting for approximately 2.8 million deaths annually [2], and this escalating prevalence of obesity has been closely paralleled by an increase in NCDs associated with elevated BMI, such as diabetes and cardiovascular diseases ([3, 4]). Negative emotional eating (EE), defined as the tendency to consume food in response to negative emotions such as stress, has been associated with higher body mass index (BMI) and adverse physical and mental health outcomes [5–7]. These negative health consequences highlight the importance of negative EE as a behavioral risk factor contributing to the global burden of disease. Excessive caloric intake is associated with weight gain, thereby understanding the risk factors of negative EE is crucial.

Chronic stress activates the hypothalamic-pituitary-adrenal axis, elevating cortisol levels that drive the consumption of energy-dense “comfort foods” as a coping mechanism. This stress-induced eating behavior increases caloric intake and fat accumulation, contributing to obesity and metabolic disorders [8]. Given that excessive caloric intake remains a key driver of weight gain, understanding the specific risk factors that contribute to overeating, including negative EE, is crucial for informing more effective prevention and intervention strategies aimed at curbing the global rise in obesity and related NCDs. Despite its growing recognition as a behavioral risk factor, negative EE differs from more established eating disorders such as binge eating disorder in that it lacks formal diagnostic criteria and is not included in either the Diagnostic and Statistical Manual of Mental Disorders, Fifth Edition (DSM-V), or the International Classification of Diseases, Eleventh Revision (ICD-11) [9, 10]. To address this gap, several validated instruments, including the Three-Factor Eating Questionnaire (TFEQ), Dutch Eating Behavior Questionnaire (DEBQ), Emotional Eater Questionnaire (EEQ), and Emotional Eating Scale (EES), have been developed to assess negative EE [11–14]. Nevertheless, the use of differing definitions and measurement tools across studies has led to substantial variability in reported prevalence rates, which are further influenced by cultural, social, and methodological factors.

Research on negative EE to date has predominantly focused on adolescents and young females, often utilizing small, non-representative samples or on clinical or psychosocial outcomes. For instance, the prevalence of high EE has been reported as 12.4% among young Saudi females and 14.8% among Chinese undergraduates [15, 16]. Furthermore, many studies have been conducted in obese or psychiatric populations, often with limited sample sizes or with an emphasis on physical or psychosocial associations rather than prevalence itself [17–19]. Data on the prevalence of negative EE among males and older populations remain scarce This gap is particularly concerning given that middle-aged adults are at increased risk for obesity-related NCDs, yet information on negative EE in this demographic is limited [20]. Moreover, middle-aged adults may be especially susceptible to negative EE due to unique life stressors and transitions experienced during this period, such as career demands, family responsibilities, and age-related health changes. The paucity of research in this area, combined with limited access to appropriate diagnosis and treatment, suggests that negative EE may be an under-examined health issue among middle-aged populations globally. Consequently, there is a pressing need for epidemiological data on negative EE to identify at-risk subgroups and facilitate the implementation of prevention and early intervention strategies before the onset of advanced NCDs.

To date, no systematic reviews have synthesized the global prevalence of negative EE in middle-aged adults. To address this gap, the present study systematically reviews the literature across WHO regions, focusing specifically on middle-aged populations. By evaluating the impact of demographic and methodological factors, this review aims to pool prevalence estimates of negative EE among middle-aged adults, thereby providing critical evidence to inform targeted public health strategies and clinical interventions.

## Methods

### Study selection

This systematic review and meta-analysis was conducted to estimate the prevalence of negative EE among middle-aged adults. The methodology was guided by Arksey and O’Malley’s six-stage framework for systematic reviews [21], with adaptations as appropriate for prevalence synthesis. All aspects of the review, including development of the research question, search strategy, eligibility criteria, data management, and analysis plan, were finalized through consensus among all authors. The review process adhered to the Preferred Reporting Items for Systematic Reviews and Meta-Analyses (PRISMA) guidelines [22]. Studies were eligible for inclusion if they: (a) reported on adults aged 35–64 years, (b) were published from 2000 onwards in peer-reviewed journals, (c) were written in English, and (d) assessed negative EE prevalence using a validated instrument. Exclusion criteria comprised animal studies, editorials, case reports, newspaper articles, other inapplicable designs, and studies with non-generalizable patient populations, such as individuals with chronic diseases or those recruited from specific occupational settings. Two independent reviewers screened studies at each stage (title, abstract, full text), with discrepancies resolved through discussion to ensure consensus. The 35–64 age range was defined as middle age based on Medley’s life stage framework [23], and country income levels were classified using the World Bank’s income categorization system [24].

### Literature search

A comprehensive literature search was conducted across five major databases: Medline (Ovid), Embase (Ovid), PsycINFO (Ovid), Web of Science Core Collection, and Google Scholar. The inclusion of PsycINFO allowed for targeted retrieval of studies in behavioral sciences and mental health, which are pertinent to negative EE [25]. Boolean operators and wildcard characters were employed to combine search terms related to negative EE and middle-aged populations. The first 200 relevant Google Scholar results were screened to supplement database searches. The detailed search strategy and keyword combinations are presented in Appendix 1.

### Data analysis

References were managed using EndNote X9, and article screening was facilitated by Covidence software to ensure accurate duplicate removal and eligibility tracking ([26, 27]). After initial title and abstract screening, the full texts of potentially eligible studies were retrieved. Where necessary, authors were contacted to obtain missing full-text articles. Key data extracted included authorship, publication year and country, study design, participant characteristics, assessment instruments, and reported prevalence rates of negative EE. Prevalence estimates were standardized based on recommended cut-off values for each instrument: mean scores >3.25 for DEBQ-EE, ≥ 3 for TFEQ-R18-EE and R21-EE, ≥ 0.69 for TFEQ-51, total scores ≥ 11 for EEQ, and >53.8 for EES [28–33]. Meta-analysis of proportions was conducted using RStudio, with pooled prevalence calculated via random-effects models [28]. Heterogeneity was assessed using the I² statistic and corresponding 95% confidence intervals, with values >50% considered indicative of significant heterogeneity [29]. Subgroup analyses were stratified by age group, sex, and assessment instrument to explore potential sources of heterogeneity. Pooled estimates for each subgroup were determined, given the influence of these variables, especially female sex, younger age, and use of the EEQ, on negative EE prevalence [13, 30].

## Result

The search identified 1,390 records, of which 26 were duplicates and subsequently removed.

**Fig. 1:**
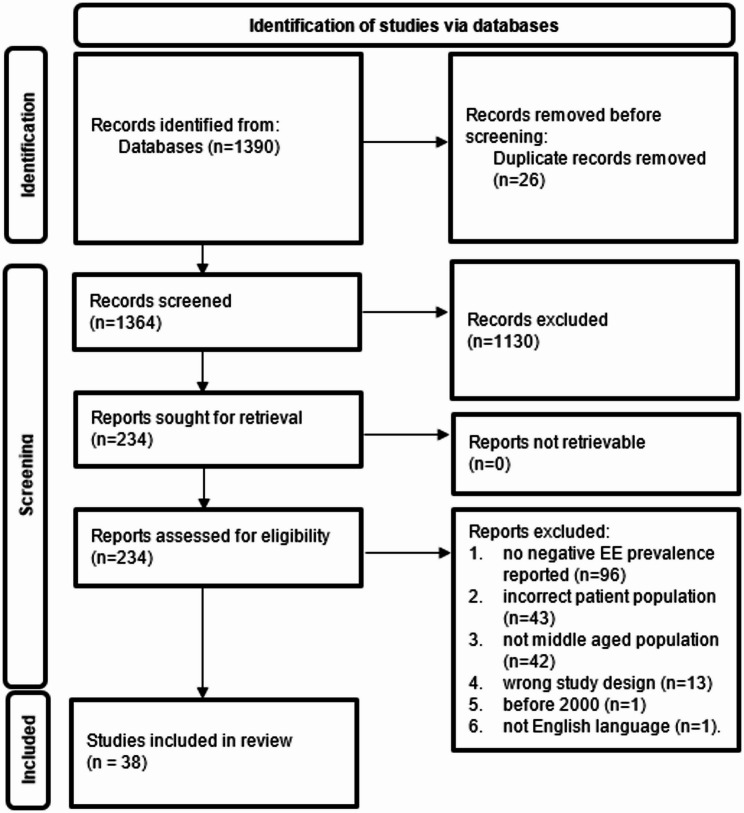
PRISMA Diagram

Following title and abstract screening, 1,130 of the remaining 1,364 articles were excluded for not meeting the inclusion criteria, leaving 234 articles for full-text review. Of these, 196 articles were excluded for the following reasons: no negative EE prevalence reported (*n* = 96), incorrect patient population (*n* = 43), not middle-aged populations (*n* = 42), inappropriate study design (*n* = 13), published before 2000 (*n* = 1), or not published in English (*n* = 1). 38 studies met all inclusion criteria and were included (See Fig. 1 for the PRISMA diagram).

The publication year and location, study design, participant characteristics, assessment instruments, and reported prevalence of the included studies are shown (see Table 1). Many of the studies were from the U.K. With fewer studies from Australia (*n* = 3), Turkey (*n* = 3), the United States (*n* = 3), Canada (*n* = 2) Italy (*n* = 2), France (*n* = 2) and one each from several other countries. According to World Bank income classification, eight studies originated from middle-income countries and thirty from high-income countries. The 38 studies included were published between 2015 and 2022 [31–68], although the publication year does not necessarily reflect the time of data collection. However, the year of publication does not reflect the year when the studies were conducted. Most studies used a cross-sectional design (*n* = 32), with the remainder being experimental (*n* = 3), secondary analyses (*n* = 2), or cohort (*n* = 1) studies. Study populations primarily included middle-aged adults from the general public or healthy-weight samples. Negative EE was assessed using DEBQ-EE (*n* = 21), TFEQ-EE (*n* = 12), EES (*n* = 3), and EEQ (*n* = 2).

**Fig. 2:**
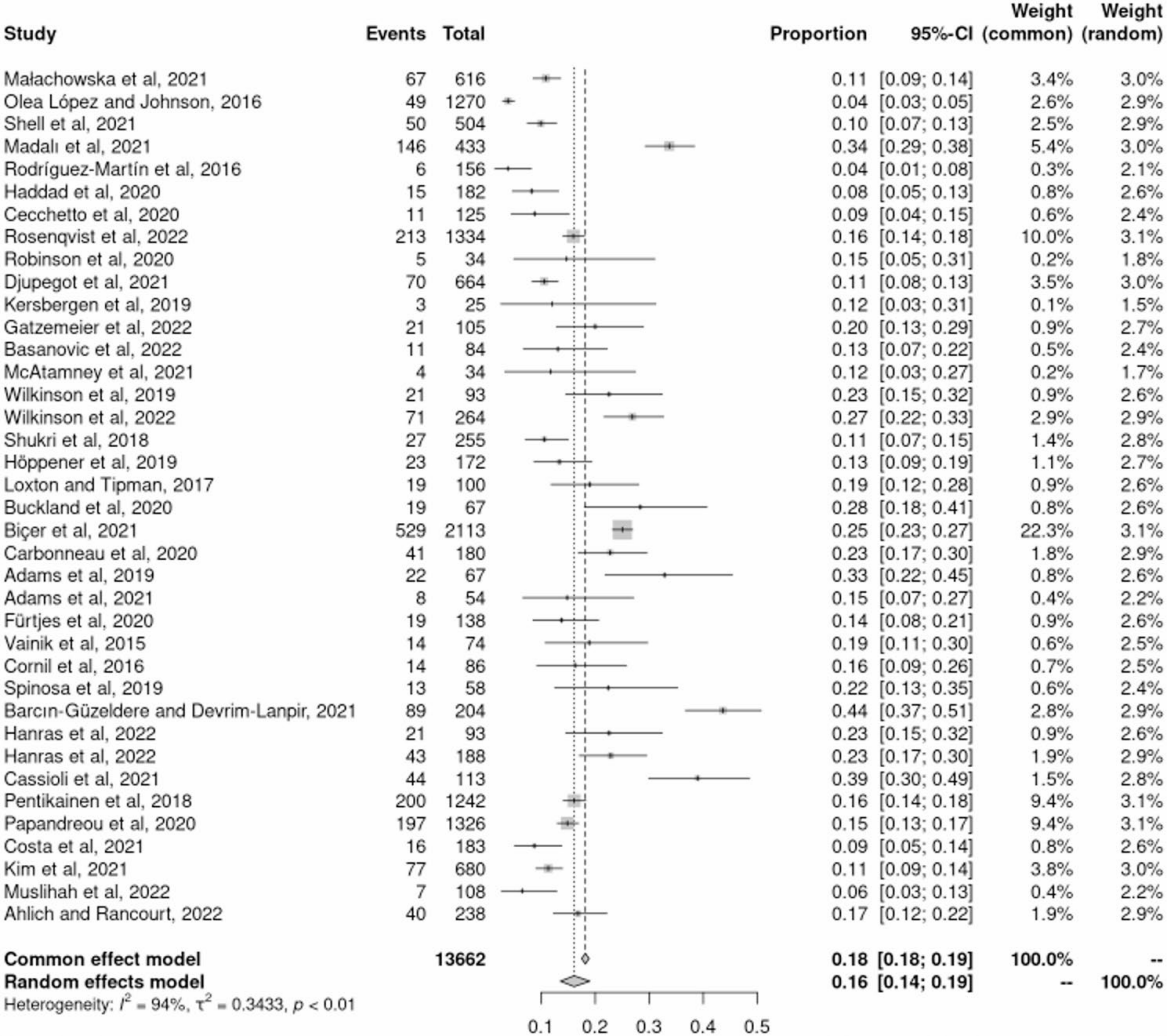
Forest plot for negative EE prevalence

Figure 2 shows that the overall pooled prevalence of negative EE among 13,662 participants across 38 studies was 16% (95% CI: 0.14–0.19), based on random-effects models. The lowest reported prevalence was 3.85% (Cuba & Italy, DEBQ-EE), while the highest was 43.63% (Turkey, EEQ). The test for heterogeneity indicated substantial statistical heterogeneity (I²=94%, *p* < 0.01).

Table 2 shows that stratification reduced I² values from 94% overall to between 0% and 86.2% within subgroups, and between-group p-values were generally non-significant. Prevalence was lower among older males and higher among younger females. The highest prevalence was observed among females aged 35–39 years measured with the EES (60%, CI: 0.35–0.81), while the lowest was among males aged 60–64 years measured with the TFEQ-EE (6%, CI: 0.03–0.15, I²: 51.4%). For studies using TFEQ-EE, prevalence was higher among younger middle-aged adults in both sexes. For instance, among females, prevalence was 26% (CI: 0.22–0.30) in the 35–39 age group versus 16% (CI: 0.12–0.21) in the 60–64 age group; among males, 19% (CI: 0.13–0.26) versus 6% (CI: 0.03–0.15) (*P* < 0.05). For DEBQ-EE, a U-shaped pattern in age was noted: among females, prevalence was 20% (35–39 years), dropped to 13% (50–54 years), and rose to 20% (60–64 years); among males, it was 12%, 9%, and 10% for the same age groups, respectively.

Table 3 shows higher negative EE prevalence in high-income countries compared to middle-income countries. There was little difference in prevalence by income level when measured with TFEQ-EE (*P* < 0.05), and there were insufficient studies for comparison using EEQ or EES. However, DEBQ-EE data showed approximately double the prevalence of negative EE in high-income countries across age groups. For example, among those aged 35–39, prevalence was 11% (CI: 0.07–0.17) in middle-income countries versus 18% (CI: 0.13–0.23, I²: 64.9%) in high-income countries. In older age groups, the prevalence remained higher in high-income settings (10%, CI: 0.07–0.16, I²: 60.2%) compared with middle-income countries (4%, CI: 0.01–0.16, I²: 0.0%).

**Table 1:** Overview of descriptive characteristics on studies included

Study	Places	Income Level	Study designs	Subject characteristics	Instrument	Event	Total Sample	Event Rate
Małachowska et al., 2021 [32]	Poland	High	Cross-sectional study	General public	DEBQ-EE > 3.25	67	616	10.88%
Olea López and Johnson, 2016 [33]	United Kingdom	High	Cross-sectional study	General public	DEBQ-EE > 3.25	49	1270	3.86%
Shell et al., 2021 [34]	United States	High	Cross-sectional study	General public	TFEQ-51-EE ≥ 0.69	50	504	9.92%
Madalı et al., 2021 [35]	Turkey	Middle	Cross-sectional study	General public	EEQ ≥ 11	146	433	33.72%
Rodríguez-Martín et al., 2016 [36]	Cuba & Italy	Middle	Cross-sectional study	Normal weight individuals	DEBQ-EE > 3.25	6	156	3.85%
Haddad et al., 2020 [37]	Lebanon	Middle	Cross-sectional study	General public	EES > 53.8	15	182	8.24%
Cecchetto et al., 2020 [38]	Italy	High	Cohort study	General public	DEBQ-EE > 3.25	11	125	8.80%
Rosenqvist et al., 2022 [31]	Finland	High	Cross-sectional study	42-year-old general public	TFEQ-R18-EE ≥ 3	213	1334	15.97%
Robinson et al., 2020 [39]	United Kingdom	High	Secondary analysis	General public	DEBQ-EE > 3.25	5	34	14.71%
Djupegot et al., 2021 [40]	Denmark	High	Cross-sectional study	General public	TFEQ-R18-EE ≥ 3	70	664	10.54%
Kersbergen et al., 2019 [41]	United Kingdom	High	Experimental study	General public	TFEQ-R18-EE ≥ 3	3	25	12.00%
Gatzemeier et al., 2022 [42]	United Kingdom	High	Cross-sectional study	General public	DEBQ-EE > 3.25	21	105	20.00%
Basanovic et al., 2022 [43]	Australia	High	Experimental study	General public	DEBQ-EE > 3.25	11	84	13.10%
McAtamney et al., 2021 [44]	United Kingdom	High	Cross-sectional study	General public	EES > 53.8	4	34	11.76%
Wilkinson et al., 2019 [45]	United Kingdom	High	Secondary analysis	General public	TFEQ-R18-EE ≥ 3	21	93	22.58%
Wilkinson et al., 2022 [46]	United Kingdom	High	Cross-sectional study	General public	TFEQ-R18-EE ≥ 3	71	264	26.89%
Shukri et al., 2018 [47]	Malaysia	Middle	Cross-sectional study	General public	DEBQ-EE > 3.25	27	255	10.59%
Höppener et al., 2019 [48]	The Netherlands	High	Cross-sectional study	General public	DEBQ-EE > 3.25	23	172	13.37%
Loxton and Tipman, 2017 [49]	Australia	High	Cross-sectional study	Female general public	DEBQ-EE > 3.25	19	100	19.00%
Buckland et al., 2020 [50]	United Kingdom	High	Cross-sectional study	General public	TFEQ-R18-EE ≥ 3	19	67	28.36%
Biçer et al., 2021 [51]	Turkey	Middle	Cross-sectional study	General public	TFEQ-R21-EE ≥ 3	529	2113	25.04%
Carbonneau et al., 2020 [52]	Canada	High	Cross-sectional study	Mothers	TFEQ-R18-EE ≥ 3	41	180	22.78%
Adams et al., 2019 [53]	United Kingdom	High	Cross-sectional study	General public	DEBQ-EE > 3.25	22	67	32.84%
Adams et al., 2021 [54]	United Kingdom	High	Cross-sectional study	Healthy-weight sample	DEBQ-EE > 3.25	8	54	14.81%
Fürtjes et al., 2020 [55]	Germany	High	Cross-sectional study	Female general public	TFEQ-R18-EE ≥ 3	19	138	13.77%
Vainik et al., 2015 [56]	Canada	High	Experimental study	Female general public	DEBQ-EE > 3.25	14	74	18.92%
Cornil et al., 2016 [57]	United States	High	Cross-sectional study	General public	DEBQ-EE > 3.25	14	86	16.28%
Spinosa et al., 2019 [58]	Australia	High	Cross-sectional study	General public	DEBQ-EE > 3.25	13	58	22.41%
Barcın-Güzeldere and Devrim-Lanpir, 2021 [59]	Turkey	Middle	Cross-sectional study	General public	EEQ ≥ 11	89	204	43.63%
Hanras et al., 2022 [60]	France	High	Cross-sectional study	General public	DEBQ-EE > 3.25	21	93	22.58%
Hanras et al., 2022 [61]	France	High	Cross-sectional study	General public	DEBQ-EE > 3.25	43	188	22.87%
Cassioli et al., 2021 [62]	Italy	High	Cross-sectional study	General public	EES > 53.8	44	113	38.94%
Pentikainen et al., 2018 [63]	Finland & Germany	High	Cross-sectional study	General public	TFEQ-R18-EE ≥ 3	200	1242	16.10%
Papandreou et al., 2020 [64]	Spain & Greece	High	Cross-sectional study	General public	DEBQ-EE > 3.25	197	1326	14.86%
Costa et al., 2021 [65]	Brazil	Middle	Cross-sectional study	General public	TFEQ R21-EE ≥ 3	16	183	8.74%
Kim et al., 2021 [66]	Korea	High	Cross-sectional study	General public	DEBQ-EE > 3.25	77	680	11.32%
Muslihah et al., 2022 [67]	Indonesia	Middle	Cross-sectional study	General public	DEBQ-EE > 3.25	7	108	6.48%
Ahlich and Rancourt, 2022 [68]	United States	High	Cross-sectional study	General public	DEBQ-EE > 3.25	40	238	16.81%

**Table 2:** Age-, sex- and instrument- stratified subgroup analysis using random effects model

	Female					Male				
	Number of studies	Pooled prevalence estimates	95% CI	I^2^	Between group *p*-value	Number of studies	Pooled prevalence estimates	95% CI	I^2^	Between group *p*-value
TFEQ-EE					**< 0.001**					0.214
Age group: 35–39	10	26%	0.22–0.30	13.8%		8	19%	0.13–0.26	33.6%	
40–44	11	25%	0.21–0.29	47.5%		7	12%	0.06–0.22	81.5%	
45–49	10	21%	0.16–0.27	39.8%		8	12%	0.07–0.21	38.0%	
50–54	7	18%	0.11–0.28	73.7%		5	12%	0.08–0.19	2.2%	
55–59	8	15%	0.12–0.20	12.8%		5	14%	0.07–0.25	50.4%	
60–64	7	16%	0.12–0.21	0.0%		4	6%	0.03–0.15	51.4%	
DEBQ-EE					0.187					0.263
Age group: 35–39	21	20%	0.15–0.26	55.0%		14	12%	0.08–0.17	19.0%	
40–44	19	20%	0.15–0.27	61.7%		11	15%	0.07–0.32	86.2%	
45–49	16	20%	0.15–0.26	50.8%		10	12%	0.08–0.16	6.2%	
50–54	18	13%	0.09–0.19	43.1%		9	9%	0.06–0.13	0.0%	
55–59	17	12%	0.08–0.29	50.6%		6	6%	0.04–0.10	0.0%	
60–64	12	20%	0.11–0.34	74.4%		8	10%	0.05–0.21	55.3%	
EEQ					0.829					0.594
Age group: 35–39	2	40%	0.27–0.55	62.0%		2	38%	0.22–0.57	17%	
40–44	2	44%	0.22–0.68	78.1%		2	35%	0.15–0.62	42%	
45–49	2	46%	0.35–0.57	0.0%		2	36%	0.24–0.50	0%	
50–54	2	38%	0.26–0.51	0.0%		2	22%	0.13–0.36	3%	
55–59	2	40%	0.23–0.58	0.0%		1	24%	0.11–0.44	--	
60–64	2	57%	0.29–0.81	0.0%		1	38%	0.13–0.72	--	
EES					0.460					0.877
Age group: 35–39	1	60%	0.35–0.81	--		2	25%	0.10–0.51	0%	
40–44	1	50%	0.27–0.73	--		2	23%	0.08–0.53	0%	
45–49	2	31%	0.09–0.68	81.8%		2	16%	0.03–0.50	16%	
50–54	3	26%	0.08–0.59	57.0%		1	7%	0.01–0.37	--	
55–59	2	40%	0.19–0.65	0.0%		2	19%	0.05–0.52	0%	
60–64	1	25%	0.06–0.62	--		1	22%	0.06–0.58	--	

**Table 3:** Age-, income- and instrument- stratified subgroup analysis using random effects model

	Middle					High				
	Number of studies	Pooled prevalence estimates	95% CI	I^2^	Between group *p*-value	Number of studies	Pooled prevalence estimates	95% CI	I^2^	Between group *p*-value
TFEQ-EE					0.060					**0.047**
Age group: 35–39	2	23%	0.15–0.33	56.8%		8	24%	0.19–0.30	44.5%	
40–44	1	27%	0.23–0.31	--		10	19%	0.15–0.24	58.8%	
45–49	2	19%	0.07–0.41	76.6%		8	17%	0.12–0.22	38.5%	
50–54	2	13%	0.02–0.47	71.6%		8	12%	0.07–0.21	74.1%	
55–59	2	14%	0.09–0.22	0.0%		6	15%	0.10–0.22	65.8%	
60–64	1	12%	0.05–0.28	--		6	13%	0.08–0.20	64.1%	
DEBQ-EE					0.456					0.070
Age group: 35–39	3	11%	0.07–0.17	0.0%		18	18%	0.13–0.23	64.9%	
40–44	2	11%	0.06–0.2	0.0%		17	19%	0.13–0.27	79.6%	
45–49	3	6%	0.03–0.14	11.8%		16	17%	0.13–0.22	54.6%	
50–54	2	7%	0.03–0.15	0.0%		16	11%	0.08–0.15	40.1%	
55–59	2	4%	0.01–0.16	0.0%		15	10%	0.07–0.16	60.2%	
60–64	1	33%	0.04–0.85	--		13	15%	0.09–0.26	77.9%	
EEQ					0.439					--
Age group: 35–39	2	40%	0.25–0.56	79.2%		--	--	--	--	
40–44	2	41%	0.19–0.67	86.5%		--	--	--	--	
45–49	2	42%	0.34–0.51	0.0%		--	--	--	--	
50–54	2	30%	0.21–0.41	19.7%		--	--	--	--	
55–59	2	31%	0.20–0.44	0.0%		--	--	--	--	
60–64	2	43%	0.25–0.64	0.0%		--	--	--	--	
EES					0.496					0.696
Age group: 35–39	1	8%	0.03–0.22	--		1	50%	0.29–0.71	--	
40–44	1	4%	0.01–0.16	--		1	47%	0.27–0.69	--	
45–49	1	15%	0.07–0.28	--		1	47%	0.27–0.69	--	
50–54	1	6%	0.02–0.22	--		2	30%	0.16–0.49	0.0%	
55–59	1	7%	0.01–0.37	--		2	35%	0.18–0.57	0.0%	
60–64	--	--	--	--		2	37%	0.07–0.81	42.3%	

## Discussion

This meta-analysis offers the first comprehensive and up-to-date estimate of the prevalence of negative EE among middle-aged adults, synthesizing findings from 38 studies published between 2015 and 2022. Our pooled prevalence of 16% indicates that negative EE is a widespread concern in this demographic, affecting approximately one in six adults globally. This prevalence highlights the need to recognize negative EE not only as a phenomenon associated with adolescence or young adulthood, but as a persistent behavioral risk factor in midlife, a period often overlooked in eating behavior research.

Subgroup analyses show marked disparities across age, sex, socioeconomic context, and assessment approach. Prevalence peaked among females aged 35–39 years, especially when measured with the EES, and was lowest among males aged 60–64 years when assessed with the TFEQ-EE subscale. The observation that EES and EEQ instruments yield higher prevalence rates than other tools suggests that instrument sensitivity, item content, and cut-off thresholds may shape prevalence estimates, highlighting a need for consensus and standardization to enable valid cross-study comparisons and reliable trend monitoring. Our results show that negative EE is more prevalent in high-income countries than in middle-income. This pattern shows greater availability of highly palatable, energy-dense foods, heightened psychosocial stress, and shifting cultural norms associated to food and emotion in affluent societies. Middle-aged females in high-income contexts may be particularly vulnerable, given increased exposure to workplace stress, caregiving responsibilities, and sociocultural pressures regarding body image. These findings are consistent with broader evidence associating socioeconomic status and sex with negative EE patterns and obesity risk [15–20]. Prior meta-analyses have either focused on negative EE or specific age groups such as adolescents or older adults, the middle-aged population is underrepresented [69–71]. Our study provides a robust, conservative prevalence estimate and mitigates potential publication bias. Given that dietary risk factors are implicated in over 11 million deaths and 255 million DALYs globally each year [72], and that diet-related healthcare costs are substantial even in high-income countries [73], identifying and addressing behavioral contributors such as negative EE in midlife is imperative for effective NCDs prevention.

Routine screening for negative EE should be considered within primary care settings, especially for younger middle-aged females and those in high-income environments. Early identification and referral to evidence-based behavioral interventions may help reduce the risk of obesity and associated NCDs [74], while also improving mental well-being and quality of life. Integrating negative EE assessment into broader health promotion strategies could enhance their reach and effectiveness. Longitudinal studies are also needed to elucidate the temporal dynamics associated with negative EE, mental health, and physical outcomes. Incorporating culturally specific variables and exploring the impact of social norms, stigma, and resilience factors could further clarify the determinants of negative EE and inform more equitable intervention strategies. Another noteworthy point is the potential associations of negative EE with mental health conditions such as depression and anxiety, which are prevalent in midlife and may exacerbate maladaptive eating behaviors [75–78]. Investigating these associations in future studies could provide valuable insights for integrative approaches to prevention and care.

Despite the strengths of this meta-analysis, several limitations warrant discussion. Substantial heterogeneity was observed, likely reflecting differences in assessment instruments, cut-off values, and sampling strategies across studies. Most subgroup I² values indicated moderate to high heterogeneity, and the small number of studies in some categories limited the feasibility of meta-regression to fully explore sources of variability. The paucity of studies from low- and lower-middle-income countries also limits the generalizability of our findings, and underscores the need for more representative, culturally sensitive research. Additionally, most included studies were cross-sectional, precluding conclusions about causal relationships between negative EE and health outcomes. Future research should quantify the association between negative emotional eating and BMI/obesity through effect size estimates, as current data are insufficient to support meta-analytic synthesis. Future studies should explore emotional eating across a broader spectrum of emotions, including those with positive valence, and examine how the type and severity of EE influence BMI, which may inform tailored intervention strategies.

## Conclusion

Negative EE is a prevalent and consequential issue among middle-aged adults, particularly females and those in high-income countries. These findings highlight the importance of standardized assessment, targeted screening, and culturally informed interventions to address this behavioral risk factor. By prioritizing research and health system responses in this area, there is significant potential to improve population health, reduce obesity and NCDs risk, and lower healthcare costs worldwide.

## Data Availability

No datasets were generated or analysed during the current study.

## Appendix 1

### Searching strategies

Ovid - Medline, Embase, PsycInfo: 1183 results.

1. (emotional adj2 eating).ti, ab, kw.
2. (three-factor adj2 eating adj2 questionnaire).ti, ab, kw.
3. (three adj2 factor adj2 eating adj2 questionnaire).ti, ab, kw.
4. (dutch adj2 eating adj2 behavior adj2 questionnaire).ti, ab, kw.
5. (emotional adj2 eater).ti, ab, kw.
6. adolescent*.ti, ab, kw.
7. teen*.ti, ab, kw.
8. student*.ti, ab, kw.
9. (young adj2 adult*).ti, ab, kw.
10. child*.ti, ab, kw.
11. 6 or 7 or 8 or 9 or 10.
12. 1 or 2 or 3 or 4 or 5 not 11.
13. (animal studies or animals, laboratory or experimental animal or animal experiment or animal model or rodentia or rodents or rodent).sh.
14. (editorial or historical article or anecdote or comment or commentary or note or case report* or case study or newspaper article or news or letter*).pt.
15. 13 or 14.
16. 8 not 15.
17. limit 16 to english language.
18. limit 17 to human.
19. limit 18 to yr="2000 -Current”.
20. remove duplicates from 19.

Web of Science: 7 results.

(TI=(emotional eating) OR AB=(emotional eating)) AND KP=(emotional eating).

Google scholar - first 200 results.

emotional eating AND adult.
